# Long-term intake of miso soup decreases nighttime blood pressure in subjects with high-normal blood pressure or stage I hypertension

**DOI:** 10.1038/s41440-019-0304-9

**Published:** 2019-08-02

**Authors:** Hiroaki Kondo, Hiroe Sakuyama Tomari, Shoko Yamakawa, Manabu Kitagawa, Minami Yamada, Seiki Itou, Tetsuro Yamamoto, Yoshio Uehara

**Affiliations:** 1 Meiseikai Higashi Shinjuku Clinic, 3F Daito-bldg., 1-11-3 Okubo, Shinjuku-Ku, 169-0072 Japan; 2 Division of Clinical Nutrition, Faculty of Home Economics, Kyoritsu Women’s University, Tokyo, Japan; 3 Marukome Co., Ltd, Nagano, Japan; 4 TTC Co., Ltd, Tokyo, Japan; 5 Food Therapeutic Lab, Tokyo, Japan

**Keywords:** ACE, Clinical study, Miso, Nighttime blood pressure, Salt

## Abstract

The present study aimed to investigate the effects of the combination of Marukome Nenrin miso, which has natriuretic effects, and Marukome MK-34-1 miso, which has potent angiotensin converting enzyme inhibitory effects, on blood pressure (BP) in humans. A total of 40 subjects aged 40–69 years with high-normal BP or stage I hypertension were randomly assigned to two groups: 1) the miso group (32 g 2:1 w/w Nenrin and MK-34-1 with 3.8 g salt/day) or 2) the control soy food group (14.4 g soy food with 0.2 g salt/day). The levels of major nutrients were equal in the miso and control food servings, except for the fiber and Na levels, which were higher in the miso food serving. Daytime and nighttime BP were measured with an automated BP monitor. Compared with the soy food intake, miso intake for 8 weeks did not affect daytime clinical BP but significantly decreased nighttime BP without affecting pulse rate (PR). Moreover, miso shifted the nighttime BP profile to lower levels than those at baseline. Soy food intake did not change the nighttime BP profile after 8 weeks. Miso intake also tended to reduce nighttime BP in a subgroup with stage 1 hypertension compared with the results of the soy food group participants and shifted the nighttime BP profile toward lower levels than those recorded at baseline. Miso intake did not influence lipid or glucose metabolism. In conclusion, this is the first report showing that miso reduces nighttime BP in humans. Miso may do so by shrinking the fluid spaces in the body and/or deactivating the adrenergic nervous system.

## Introduction

Common miso is a traditional Japanese food routinely consumed by Japanese people in soup. In Japan, 80–90% of common miso is produced from soybeans with malted rice and salt, i.e., rice-miso. One serving traditional (common) miso soup usually contains 1–2 g of salt. It is widely recognized that a high salt intake is often associated with increased blood pressure (BP) and risk of cardiovascular events and stroke [1, 2]. Therefore, common miso soup consumption is thought to underlie the high incidence of salt-sensitive hypertension in Japan. This long-held assumption is, however, contradicted by a recent 5-year cross-sectional observational study showing no association between the frequency of common miso soup intake and BP. In that study, daily salt intake (self-reported) correlated with the frequency of common miso consumption but not with BP: 7.6 ± 2.8 vs 11.3 3 ± 2.9 g salt/day (*P* < 0.001) in the normal BP group and 7.7 ± 2.7 vs 11.3 ± 4.0 g salt/day (*P* < 0.001) in the high-normal BP group for low vs high general miso intake, respectively [3]. Moreover, in a recent interventional trial, two daily servings of common miso soup (3.8 g salt per day) for 3 months did not affect daytime BP in untreated subjects with normotension or stage I hypertension [4]. These findings clearly indicate that high salt intake from common miso does not increase BP in humans.

When the salt contents are equal, BP is lower when common miso soup is consumed vs saline consumption. Hence, common miso intake may actually decrease BP, as shown in previous animal studies. The suggested mechanisms include increased natriuresis and vasodilation, both of which are thought to account for the attenuated salt-induced hypertension in Dahl salt-sensitive (Dahl S) rats fed commercially available common miso produced with fermented rice [5, 6]. However, we have found that general miso manufactured with a Koji starter (molted wheat) and soybeans (wheat-miso) does not induce natriuresis in Dahl S rats (unpublished data). If natriuresis reduces BP in people with long-term common miso intake, shrinkage of the fluid spaces in the body presumably activates the renin-angiotensin system (RAS), which may counteract the antihypertensive effects of miso.

Conversely, inhibiting RAS activation may enhance the antihypertensive effects of common miso. Using Koji species different from those used for common miso, we manufactured a new miso product containing a potent angiotensin-converting enzyme (ACE) inhibitor (MK-34-1 miso; Marukome Co., Ltd., Nagano, Japan) [7]. To potentially strengthen its antihypertensive effects, we combined it with common miso (Nenrin miso; Marukome Co., Ltd.), which has natriuretic effects [8]. Moreover, we found that a 2:1 (w/w) combination of Nenrin and MK-34-1 miso decreased BP in Dahl S rats to a greater extent than did Nenrin miso alone or a 1:1 (w/w) combination (unpublished data).

A previous animal study demonstrated the benefits of a combination of Nenrin and MK-34-1 miso on BP. In humans, we found no difference in BP between individuals consuming Nenrin and those consuming MK-34-1 miso soup (two servings per day for 3 months) [4]. The present study explored whether long-term intake of a 2:1 (w/w) combination of Nenrin and MK-34-1 miso decreased BP in humans relative to the effects of a control (soy food without added salt). This is the first study in humans in which the salt content of the control was not adjusted to that of miso.

## Methods and subjects

### Study design

The present study was performed according to the 2014 Guidelines for the Management of Hypertension (Japanese Society of Hypertension, JSH). This was a randomized, double-blind, placebo-controlled trial. We screened 349 apparently healthy men and women aged 40 to 69 years. The subjects had BPs ranging from high-normal (130–139/85–89 mmHg) to untreated stage I hypertension (140–159/90–99 mmHg) according to the 2014 criteria of the JSH. None of the patients were being treated for any other disease, and all were screened for secondary hypertension, cardiovascular and metabolic diseases, food allergies, and other serious conditions. Screening procedures included a physical examination, routine laboratory tests, urinalysis, and a clinical history review. Eligible subjects provided informed consent before entering the study. Forty subjects were randomly allocated to the soy food (control) group (*n* = 20) or the miso group (*n* = 20). All subjects were followed for 8 weeks. During the study, all subjects were instructed to adhere to their usual diets, exercise patterns, and lifestyles. Supplements or any other foods specially designed for health purposes were not permitted. Subjects visited the clinic for examinations before entry (baseline), at week 4 and at week 8 after consuming the soy food or the miso. Casual BP was determined at each visit, and blood samples were obtained for analysis at baseline and at week 8. Moreover, nighttime BP was measured at baseline and at week 8 using automatic BP monitoring devices.

### Miso and soy food

The awase (test) miso consisted of a 2:1 (w/w) combination of Nenrin miso [12% NaCl (w/w)] and MK-34-1 miso [12% NaCl (w/w)]. Nenrin miso is a commercially available rice miso used in daily life that is manufactured using soybeans, rice, salt, and an *Aspergillus oryzae* Koji starter. MK-34-1 miso is manufactured using soybeans, rice, salt, and a Koji starter and is not patented [5]. The MK-34-1 miso has 10 times more ACE inhibitory activity than the Nenrin miso (IC_50_ = 0.23 and 2.5 mg/dL, respectively). One serving of awase miso soup contained 16 g of awase miso (1.9 g salt) and 160 mL of hot water.

The control product (soy food) was manufactured using powdered soybeans and rice with no added salt. Hence, the salt content of the soy food was approximately 20-fold lower than that of awase miso. One serving of soy food contained 7.2 g of the mixed powder (0.1 g salt) and 160 mL of hot water.

The subjects received two servings of awase miso soup or soy food per day: one at breakfast and one at supper, for 2 months. There were no differences in the major nutrient contents of the awase miso and the soy food (Table 1). The cumulative salt intake over the 8 weeks was 212 g for the awase miso group and 11 g for the soy food group.

**Table 1:** Contents of soy food and miso

Nutrition	unit	Soy food	Miso
Energy	kcal	62	61
Protein	gram	4.1	3.7
Lipid	gram	1.8	1.9
Carbohydrate	gram	7.3	7.4
Fiber	gram	1.2	2.4
Salt*	gram	0.2	3.8
Potassium	gram	0.154	0.158

The values were contents in two servings of soy food (14.4 g) and miso (32 g). Soy food was manufactured so that the contents were matched to those of miso. However, salt was not added to the soy food. *Na content was expressed as equivalent to salt

### Blood pressure measurement

BP was measured using an OMRON HEM-7252G-HP automatic BP monitor (Tokyo, Japan) according to the guidelines for home BP monitoring (Japanese Society of Hypertension) [9]. Daytime casual BP measurements were performed twice at the clinic between 0930 and 1230 hours before and 4 and 8 weeks after study entry. Before measurement, the subjects rested for 20 min in a sitting position in a warm and quiet room. Trained medical staff who were blinded to the allocation measured BP and pulse rates (PRs) more than two times at 2-min intervals. Stable and consecutive measurements were averaged.

For nighttime blood pressure monitoring at home, an automatic BP monitor recorded the BP and PR before bedtime, at 0200 and 0400 hours, and upon waking for 2 consecutive days before and 8 weeks after study entry. The measurements were averaged for analysis.

### Determination of blood parameters

Blood and biochemical parameters were determined using an automatic Sysmex XE-2100 differential analyzer (LSI Medience, Tokyo, Japan) and a LABOSPECT 008 α analyzer (Hitachi High-Technologies Corporation, Tokyo, Japan). Blood sugar was determined using the GK-G6PD enzymatic method (JCA-BM9130, JCA-BM9030, JEOL Ltd., Tokyo, Japan). Plasma human atrial natriuretic peptide (hANP) concentrations were determined by the chemiluminescent enzyme immunoassay method.

### Ethics statement

This study was designed and performed in accordance with the Declaration of Helsinki and was approved by the clinical ethics committee of Aisei Hospital and Ueno Clinic (Tokyo, Japan). The study also followed the Ethical Guidelines for Medical and Health Research Involving Human Subjects of the Ministry of Education, Culture, Sports, Science and Technology and the Ministry of Health, Labour and Welfare (IRB12000071) and was entered into the UMIN Clinical Trials Registry (UMIN 000031764).

### Statistical analysis

We previously investigated the effects of MK-34-1 miso on blood pressures in people with stage I hypertension [4]. Using the data, we estimated the sample size required to identify differences in BP between the two groups. When the type I error is <0.05 and power > 0.7, the required sample size is 20 or more. Based on this assumption, we enrolled 20 subjects in each group.

Statistical analyses were performed using STATISTICA software (StatSoftware Inc., Palo Alto, CA, USA). Values are expressed as the mean ± standard deviation (SD). Differences were assessed by Student’s *t*-test, one-way ANOVA or repeated measures analysis of variance for parametric analysis or by nonparametric Mann–Whitney U test. We also analyzed the data using the chi-square or Spearman’s R test (MedCalc Statistical Software version 18.11.6, 2019, Ostend, Belgium). *P* values < 0.05 were considered statistically significant.

## Results

**Fig. 1:**
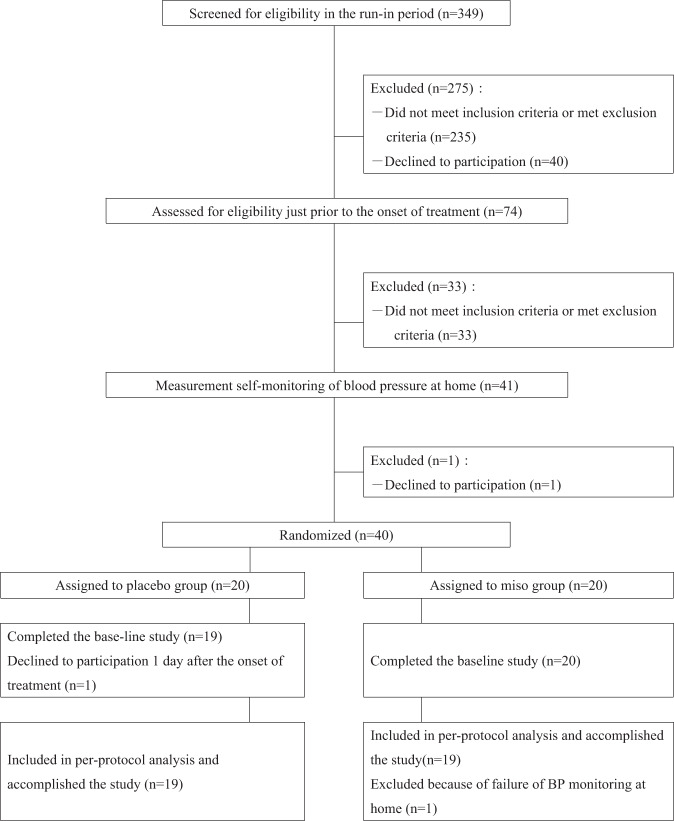
Study flow diagram. Among the 349 subjects screened, 40 were eligible for the study. Finally, 19 in the control group and 19 in the miso group were analyzed

A flow diagram of the study is shown in Fig. 1. Two subjects withdrew after group allocation, one for personal reasons and the other for failing to monitor nighttime BP because of sleep disturbance. BP profiles based on BP measurements at multiple time points were compiled for the 38 subjects in the study (19 in the control group and 19 in the miso group). The demographics of the two groups are shown in Table 2. There were no significant differences in sex, age, ratio of high-normal to stage-1 hypertensives, body weight, height or body mass index between the control and miso groups.

**Table 2:** Baseline characteristics

Parameters	Control group (*n* = 19)	Miso group (*n* = 19)	*p*-value
Men/women (number)	15/4	13/6	0.71
Age (years)	53 ± 7 (42–69)	54 ± 7 (41–68)	0.85
HN/St-1 EH (number)	5/14	4/15	1.00
Height (cm)	171.3 ± 7.7	166.4 ± 8.3	0.067
Body Weight (kg)	68.7 ± 10.3	68.6 ± 10.6	0.99
BMI (kg/m^2^)	23 ± 3	24 ± 3	0.22

HN/St-1 EH, subjects with high normal/stage 1 hypertension, *BMI* body mass index, Values are expressed as means ± standard deviation. Differences in sex were assessed using the χ^2^ test. Other differences were assessed using one-way analysis of variance (ANOVA)

### Daytime BP changes

**Fig. 2:**
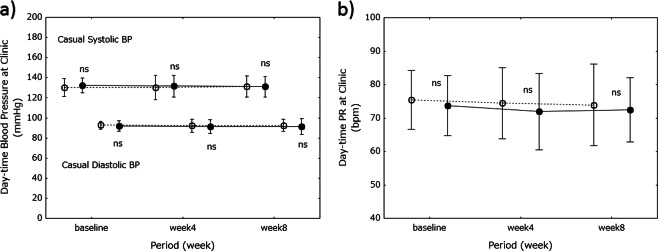
Daytime blood pressure and pulse rate at the clinic. Daytime BP measurements at the clinic are shown on graph-a. Daytime PR measurements at the clinic are shown on graph-b. Open circles, control (soy food) group; closed circles, miso group. Values are expressed as the mean±standard deviation. Differences between the groups were analyzed using Student’s *t*-test. *Ns* not significant

Daytime systolic BP (SBP) and diastolic BP (DBP) were determined at the clinic between 0930 and 1230 hours. Neither changed during the 8-week study period in the control group nor—despite the greater consumption of salt (3.8 g per day)—the miso group (Fig. 2a). There were no significant differences in BP between the groups at the times tested. Similar findings were obtained for daytime PR (Fig. 2b).

### Nighttime BP differences between the control and miso groups

**Fig. 3:**
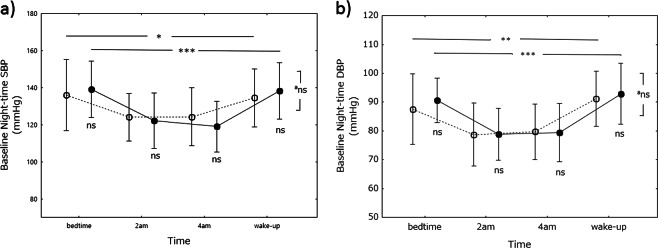
Baseline nighttime blood pressure. Nighttime systolic blood pressure (SBP) is shown on graph-a and nighttime diastolic blood pressure (DBP) on graph-b. Open circles, control (soy food) group; closed circles, miso group. Values are expressed as the mean±standard deviation. Differences in blood pressure between time points in each group and between the groups were analyzed using one-way analysis of variance and Student’s *t*-test, respectively. *P < 0.05, **P < 0.001, ***P < 0.0001. ^a^Differences in BP profile during the whole night were assessed by repeated measures analysis of variance. Ns, not significant

Nighttime SBP and DBP were determined before the study (baseline). In both groups, SBP and DBP were lower at 0200 and 0400 hours than at bedtime and waking (Fig. 3). The decrease in SBP at 0200 and 0400 hours was significant in the control (*P* < 0.05) and miso (*P* < 0.0001) groups, as was the decrease in DBP (control group, *P* < 0.01; miso group, *P* < 0.0001). However, there were no differences in either SBP or DBP between the groups at any of the times tested.

**Fig. 4:**
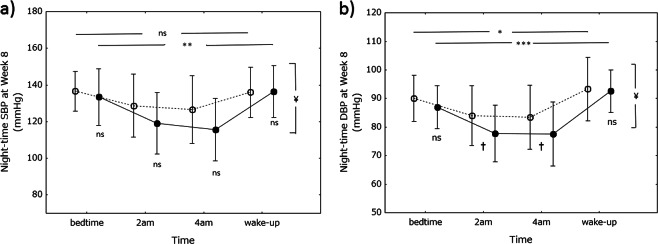
Nighttime blood pressure at week 8. Nighttime systolic blood pressure (SBP) is shown on graph-a and nighttime diastolic blood pressure (DBP) on graph-b. Open circles, control (soy food) group; closed circles, miso group. Values are expressed as the mean±standard deviation. Differences in blood pressure between time points in each group and between the groups were analyzed using one-way analysis of variance and Student’s *t*-test, respectively. *P < 0.05, **P < 0.001, ***P < 0.0001. ^†^*P* < 0.05 vs control group. Differences in BP profile during the whole night were assessed by repeated measures analysis of variance. ^¥^*P* < 0.05. *Ns* not significant

Nighttime SBP and DBP were also determined 8 weeks after study entry; both varied in the same way as baseline SBP and DBP (Fig. 4). However, the variations were significant in the miso group only. Nighttime SBP at 0200 and 0400 hours tended to be lower in the miso group than in the control group, but the differences were not statistically significant (*P* = 0.085 at 0200 hours and *P* = 0.062 at 0400 hours). However, the group difference in the nighttime SBP profile during the whole night between the control and miso groups was statistically significant (*P* < 0.05). Nighttime DBP at 0200 and 0400 hours was significantly lower in the miso group than in the control group, and the group difference in the nighttime DBP profile during the whole night between the control and miso groups was statistically significant (*P* < 0.05). There were no differences in SBP or DBP at bedtime or upon waking up between the two groups.

### Nighttime BP differences between baseline and after week 8

Differences in the BP profile during the whole night between baseline and after 8 weeks were assessed by repeated measures analysis of variance. Nighttime SBP profiles did not differ between baseline and after 8 weeks in the control group (*P* = 0.167), whereas in the miso group, the profile was significantly lower after 8 weeks than that at baseline (*P* < 0.05). The nighttime DBP profile after 8 weeks tended to be lower than that at baseline in the miso group (*P* = 0.066), but in the control group, the profile was higher after 8 weeks than at baseline (*P* < 0.05).

### Nighttime PR

**Fig. 5:**
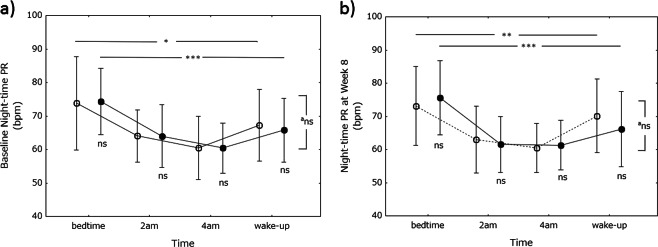
Nighttime pulse rate (PR). Baseline nighttime PR is shown on graph-a and nighttime PR at week 8 on graph-b. Open circles, control (soy food) group; closed circles, miso group. Values are expressed as the mean ±  standard deviation. Differences in PR between time points in each group and between the groups were analyzed using one-way analysis of variance and Student’s *t*-test, respectively. *P < 0.05, **P < 0.001, ***P < 0.0001. ^a^Differences in the PR profile during the whole night were assessed by repeated measures analysis of variance. *Ns* not significant

Nighttime PR was significantly lower at 0200 and 0400 hours than at bedtime and waking in the control and miso groups both before study entry and 8 weeks thereafter (Fig. 5). There were no differences in nighttime PR between the control and miso groups at the times tested. There were no differences in the PR profile during the whole night between baseline and after 8 weeks.

### BP in the stage I hypertensive subgroup

**Fig. 6:**
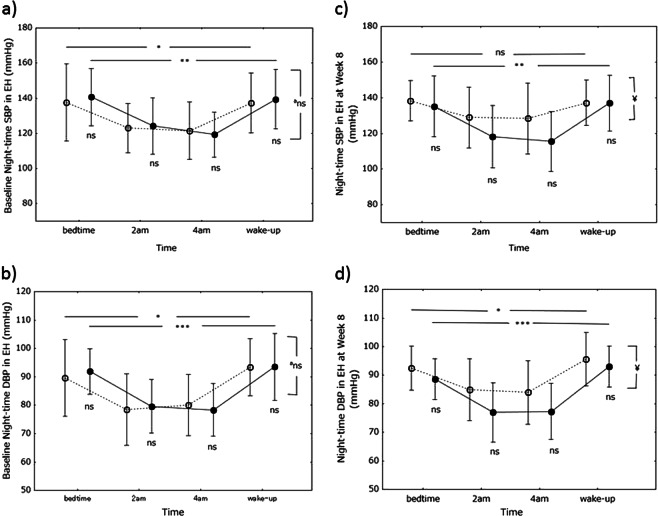
Nighttime blood pressure in subjects with stage I hypertension. Baseline nighttime systolic blood pressure (SBP) and baseline diastolic blood pressure (DBP) are shown on graph-a and graph-b, respectively. Nighttime SBP and DBP at week 8 are shown on graph-c and graph-d, respectively. Open circles, control (soy food) group; closed circles, miso group. Values are expressed as the mean ± standard deviation. Differences in blood pressure between time points in each group and between groups were analyzed using one-way analysis of variance and Student’s *t*-test, respectively. *P < 0.05, **P < 0.001, ***P < 0.0001. Differences in the BP profile during the whole night were assessed by repeated measures analysis of variance. ^¥^*P* < 0.05. *Ns* not significant

Nighttime BP was determined in the control and miso subgroups with stage 1 hypertension. There were 15 subjects in the miso subgroup and 14 in the control subgroup. Both baseline SBP and DBP profiles showed a significant U-shaped phenomenon, and there were no differences in BP at any of the times tested between the subgroups (Fig. 6). At 8 weeks, both SBP and DBP showed U-shaped profiles as did the baseline BP profiles; the SBP profile was not significantly different in the control subgroup. SBP at 0200 and 0400 hours tended to be lower in the miso group than in the control group, but the differences were not statistically significant (*P* = 0.103 at 0200 and *P* = 0.069 at 0400 hours). However, the group difference during the whole night between the control and miso groups was statistically significant (*P* < 0.05). DBP at 0200 and 0400 hours was 9.4% and 8.0% lower in the miso group than in the control group, respectively. The differences were not statistically significant (*P* = 0.052 at 0200 and *P* = 0.095 at 0400 hours). However, the group difference during the whole night between the control and miso groups was statistically significant (*P* < 0.05).

Differences in the BP profile during the whole night between baseline and after 8 weeks were assessed by repeated measures analysis of variance. In the control group, the nighttime DBP profile after 8 weeks was higher than at baseline (*P* < 0.05). In the miso group, however, the profile tended to be lower after 8 weeks than at baseline (*P* = 0.092).

**Fig. 7:**
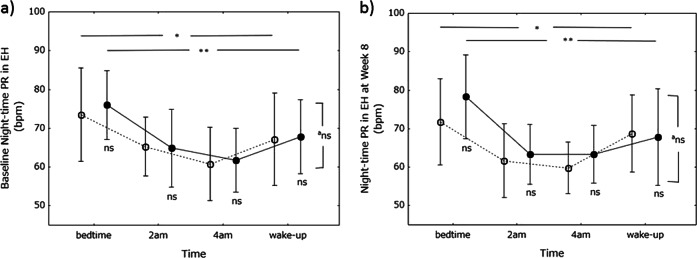
Nighttime pulse rate (PR) in subjects with stage I hypertension. Baseline nighttime PR is shown on graph-a and nighttime PR at week 8 on graph-b. Open circles, soy food (control) group; closed circles, miso group. Values are expressed as the mean ± standard deviation. Differences in PR between time points in each group and between groups were analyzed using one-way analysis of variance and Student’s *t*-test, respectively. *P < 0.05, **P < 0.001. ^a^Differences in the PR profile during the whole night were assessed by repeated measures analysis of variance. Ns not significant

There were no differences in nighttime PR between the subgroups at either baseline or 8 weeks (Fig. 7).

### Parameters related to BP control

**Fig. 8:**
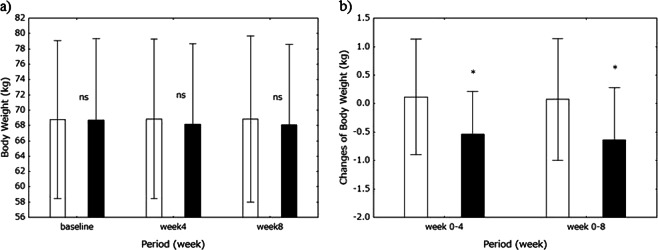
Body weight (BW) changes. Body weight is shown on graph-a and changes in body weight on graph-b. Baseline, open bars; week 4, solid bars; and week 8, filled bars. Values are expressed as the mean±standard deviation. Differences in BW between time points and between groups were analyzed using one-way analysis of variance and Student’s *t*-test, respectively. *P < 0.05 vs. the control. Ns not significant

Body weight at week 8 was 68.0 ± 10.5 kg for the miso group and 68.8 ± 10.8 kg for the control group. There were no differences in body weight, although the miso group consumed 212 g salt compared with 11 g in the control group. The changes in body weight at 4 weeks were 0.115 ± 1.016 kg for the control group and −0.536 ± 0.747 kg for the miso group (*P* < 0.05), and those at 8 weeks were 0.073 ± 1.072 kg for the control group and −0.636 ± 0.919 kg for the miso group (*P* < 0.05) (Fig. 8).

The number of white blood corpuscles at week 8 differed between the two groups but was within the normal range (Table 3). The levels of other blood components were similar in both groups at this time. Biochemical analysis showed that miso intake significantly increased low-density lipoprotein cholesterol (LDL-C) levels, which was not observed in the control group; LDL-C levels at week 8 were significantly higher in the miso group than in the control group (Table 4).

**Table 3:** Blood corpuscle analysis

Values (unit)	Sex	Group	*n*	Baseline	week 8
WBC (/µL)		Control	19	5610 ± 1446	5273 ± 1031
		Miso	19	6136 ± 1163	6052 ± 1238*
RBC (×10^4^/µL)	Male	Control	15	491 ± 38	488 ± 36
		Miso	13	502 ± 44	479 ± 43
	Female	Control	4	471 ± 34	471 ± 35
		Miso	6	467 ± 37	458 ± 39
Hb (g/dL)	Male	Control	15	14.9 ± 0.9	14.8 ± 0.6
		Miso	13	15.7 ± 0.8*	15.2 ± 0.8
	Female	Control	4	14.0 ± 0.9	13.9 ± 0.9
		Miso	6	14.0 ± 0.9	14.0 ± 1.1
Ht (%)	Male	Control	15	45.8 ± 2.2	45.6 ± 2.1
		Miso	13	48.3 ± 2.5*	46.8 ± 3.0
	Female	Control	4	43.7 ± 2.9	43.9 ± 3.0
		Miso	6	44.5 ± 2.7	43.8 ± 2.7
PLT (×10^4^/µL)		Control	19	26.1 ± 3.3	25.6 ± 4.6
		Miso	19	27.5 ± 6.4	26.9 ± 6.4

*WBC* white blood corpuscle, *RBC* red blood corpuscle, *Hb* hemoglobin, *Ht* hematocrit, *PLT* platelet. Values are expressed as mean ± standard deviation. The difference in the platelet numbers was assessed using the Mann–Whitney U test. Other differences were analyzed using an unpaired *t*-test. *P < 0.05 vs control

**Table 4:** Blood chemical analysis

Parameters (unit)	Sex	Group	*n*	Period	
				Baseline	Week 8
TP (g/dL)		Control	19	7.1 ± 0.3	7.1 ± 0.3
		Miso	19	7.4 ± 0.2**	7.3 ± 0.3†
Alb (g/dL)^*1^		Control	19	4.3 ± 0.2	4.3 ± 0.2
		Miso	19	4.5 ± 0.2*	4.5 ± 0.2*
ALP (U/L)^*1^		Control	19	207 ± 87	201 ± 68
		Miso	19	204 ± 58	200 ± 60
AST(GOT) (U/L)^*1^		Control	19	20 ± 6	20 ± 7
		Miso	19	20 ± 3	21 ± 4
ALT(GPT) (U/L)^*1^		Control	19	19 ± 9	18 ± 8
		Miso	19	21 ± 9	19 ± 6
γ-GTP (U/L)^*1^	Male	Control	15	32 ± 14	36 ± 22
		Miso	13	38 ± 21	37 ± 27
	Female	Control	4	34 ± 23	25 ± 24
		Miso	6	37 ± 30	36 ± 29
TC (mg/dL)		Control	19	207 ± 32	199 ± 36
		Miso	19	227 ± 31^†^	219 ± 26^†^
TG (mg/dL)^*1^		Control	19	89 ± 42	93 ± 36
		Miso	19	112 ± 55	104 ± 40
HDL-C (mg/dL)	Male	Control	15	65 ± 15	61 ± 11
		Miso	13	63 ± 20	58 ± 18
	Female	Control	4	77 ± 21	70 ± 25
		Miso	6	59 ± 19	55 ± 15
LDL-C (mg/dL)		Control	19	119 ± 26	113 ± 33
		Miso	19	142 ± 27*	138 ± 25*
BUN (mg/dL)		Control	19	13.1 ± 2.5	13.6 ± 3.7
		Miso	19	12.8 ± 2.7	13.4 ± 3.1
Cre (mg/dL)	Male	Control	15	0.91 ± 0.10	0.90 ± 0.08
		Miso	13	0.80 ± 0.11*	0.82 ± 0.11*
	Female	Control	4	0.69 ± 0.05	0.67 ± 0.10
		Miso	6	0.61 ± 0.03*	0.61 ± 0.03
eGFR (mL/min/1.73 m^2^)		Control	19	69.36 ± 2.09	69.92 ± 1.92
		Miso	19	79.45 ± 2.70**	77.72 ± 2.62*
CKD G1/G2/G3 (subjects)		Control	19	1/16/2	0/17/2
		Miso	19	3/15/1	3/15/1
UA (mg/dL)^*1^	Male	Control	15	5.9 ± 0.7	6.1 ± 0.6
		Miso	13	6.5 ± 0.5*	6.6 ± 0.7†
	Female	Control	4	5.1 ± 1.1	5.2 ± 0.8
		Miso	6	4.6 ± 1.2	4.7 ± 1.2
Na (mEq/L)^*1^		Control	19	141 ± 1	140 ± 1
		Miso	19	141 ± 1	141 ± 1
K (mEq/L)		Control	19	4.4 ± 0.2	4.4 ± 0.4
		Miso	19	4.3 ± 0.3	4.3 ± 0.4
Glu (mg/dL)^*1^		Control	19	92 ± 9	88 ± 6
		Miso	19	90 ± 6	87 ± 8
hANP (pg/mL)^*1^		Control	19	19.9 ± 10.4	16.3 ± 6.4
		Miso	19	13.3 ± 4.7	11.9 ± 5.9

*TP* total protein, *Alb* albumin, *ALP* alkaline phosphatase, *AST (GOT)* aspartate transaminase (glutamic-oxaloacetic transaminase), *ALT (GTP)* alanine transaminase (glutamic-pyruvic transaminase), *TC* total cholesterol, *TG* triglyceride; *HDL-C* high-density lipoprotein cholesterol, *LDL-C* low-density lipoprotein cholesterol, *BUN* blood urea nitrogen, *Cre* creatinine, *eGFR* estimated glomerular filtration rate, *CKD* stage of chronic kidney disease, *UA* uric acid, *Glu* glucose, *hANP* human atrial natriuretic peptide. Values are expressed as mean ± standard deviation. The unpaired *t*-test and Mann–Whitney U test (*1) were used to evaluate between-group differences. ^†^*P* < 0.1, *P < 0.05, **P < 0.01 vs control

Creatinine concentrations were lower in the miso group than in the control group at baseline and week 8 (Table 4). In contrast, the eGFR at baseline and at week 8 was higher in the miso group than in the control group. However, creatinine concentrations did not vary during the 8-week study period in either the control (−0.004±0.058 mg/dL) or miso (0.021 ± 0.048 mg/dL) groups. Similarly, changes in eGFR from baseline to week 8 were 0.55 ± 1.36 mL/min/1.73 m^2^ for the control group (*P* = 0.69) and −1.72 ± 1.19 for the miso group (*P* = 0.165). Moreover, the CKD stage distribution remained unchanged in the miso group, whereas one subject with grade 1 transitioned to grade 2 in the control group. The CKD staging distribution pattern was not significantly different between the two groups at baseline and week 8, as assessed by the chi-square test. There were no correlations between nighttime BP and eGFR at week 8 (*r* = −0.292, *P* = 0.075 for SBP at 0200 hours and *r* = −0.191, *P* = 0.251 for SBP at 0400 hours; *r* = −0.198, *P* = 0.234 for DBP at 0200 hours and *r* = −0.166, *P* = 0.320 for DBP at 0400 hours, assessed by Spearman R test).

## Discussion

We previously reported that commercially available common miso with fermented rice attenuates salt sensitivity in Dahl S rats [5, 6, 8, 10]. Based on the results of these studies, in which salt intake was standardized, we proposed several mechanisms through which common miso intake reduces BP in animals. Whether common miso intake also reduces BP in humans has been technically difficult to determine. To address this issue, we manufactured a new miso product (MK-34-1) with potent ACE inhibitory activity. In rats, MK-34-1 miso markedly attenuated spontaneous hypertension by inhibiting the adrenergic nervous system and the RAS [8]. In a parallel comparative interventional study in humans, the intake of MK-34-1 miso soup twice daily for 3 months significantly decreased or maintained BP during the test period [4]. Combining common miso with diuresis activators and MK-34-1 miso with RAS inhibitors may be even more effective, as may a 2:1 combination of common miso and MK-34-1 miso in Dahl S rats. Here, we explored whether Nenrin miso and MK-34-1 miso combination reduces BP in humans relative to a soy food control. The total salt intake from these foods over the 8-week study period was 212 g per subject for the miso group and 11 g per subject for the control group.

In the present study, we did not determine plasma ACE activity because of the limited blood sample due to performing blood and biochemical analyses. However, we had reported in our previous animal study that plasma ACE activity in Dahl S rats, when the rats were treated with the awase miso (2:1 w/w, Nenrin and MK-34-1 miso), did not change in comparison with the saline solution group [8]. It might be technically difficult to assess slight changes in ACE activity and angiotensin II concentrations following the administration of ACE inhibitors. The pathophysiological role of RAS following awase miso intake in the nighttime BP remains to be elucidated.

The nutrient content was similar between the soy food and miso according to the “Standard Tables of Food Composition in Japan 2015” (Ministry of Education, Culture, Sports, Science and Technology, Japan). L-arginine contents are 2.8 g/100 g in soybean and 0.48 g/100 g in rice. The daily dose of awase miso was manufactured with 9.27 g soybean and 5.73 g rice, and the daily dose of soy food was manufactured with 9.42 g soybean and 5.71 g rice. Thus, L-arginine intake was 0.287 g/day for the awase miso group and 0.291 g/day for the soy food group, suggesting that the L-arginine-nitric oxide system might not account for the nighttime BP findings in the awase miso group.

This placebo-controlled intervention study is the first to show that long-term awase miso intake does not increase daytime BP relative to a soy food control without added salt. The differences in sodium and potassium contents between soy food and awase miso might have caused a lower BP in the soy food group than in the awase miso group. The daytime BP at each visit did not differ between the miso and soy food groups. This finding may indicate that the daytime BP is actually lower in the miso group than in the control group.

More interestingly, awase miso intake (but not soy food intake) significantly decreased nighttime BP at 0200 and 0400 hours. Although we did not directly determine whether our subjects were asleep at 0200 and 0400 hours (i.e., undergoing rapid eye movement), both BP and PR were lower at these times than upon waking. Hence, they were presumably asleep when BP was recorded at these times.

Decreases in nighttime BP were not associated with increases in PR, which strongly suggests that awase miso intake dampens the baroreceptor reflex. However, there is little evidence that long-term common miso intake inhibits the adrenergic nervous system. Ito et al. reported that common miso intake attenuates hypertension caused by saline injection into the brain cavity [11]. We also reported that MK-34-1 miso attenuates spontaneous hypertension from an upregulated adrenergic nervous system [8]. However, it also increases the urinary excretion of dopamine, a catecholamine vasopressor, and there are no consistent data on the status of the sympathetic nervous system following long-term general miso intake [6]. Thus, nighttime BP reduction cannot be explained solely by the inactivation of the adrenergic nervous system. We have shown that common miso contains vasodilators, which might have mediated the nighttime BP reduction in the miso group in our study.

Body fluid metabolism also affects nighttime BP [12–15]. Diuretic intake and decreases in body fluid levels are associated with decreases in nighttime BP and the shift from nondipper to dipper BP profiles. However, we could not detect decreases in hANP in response to miso intake, probably because the values of hANP were far below the normal limit (43 pg/mL). The baseline hANP was 19.9 ± 10.4 pg/mL for the control group and 16.3 ± 6.4 pg/mL for the miso group (*P* = 0.365, Mann–Whitney U Test). The hANP at week 8 was 13.3 ± 4.7 pg/mL for the control group and 11.9 ± 5.9 pg/mL for the miso group (*P* = 0.335, Mann–Whitney U Test).

Otherwise, we found that body weight gain was lower in the miso group (Fig. 8). Thus, long-term awase miso intake may reduce BP by reducing body fluid levels [5, 6, 8]. Although we did not investigate overall food intake, there were no differences in parameters indicating nutritional status between the miso and control groups. This finding suggests that the changes in body weight were due to changes in body fluid levels, not nutritional status. In support of these findings, we have shown that long-term general miso intake increases urinary Na excretion and the fractional excretion of Na [6]. We also reported the possibility that the intake of a miso combination shrinks the fluid-filled spaces in Dahl S rats [8]. The increases in serum total protein and albumin levels observed in the present study may reflect decreases in the sizes of the body fluid spaces. To elucidate whether body fluid spaces decrease with miso intake, however, more accurate balance studies or a direct determination of the body fluid spaces are needed.

In our previous animal studies, we matched the salt concentration in common miso soup with that of a saline solution [5, 6, 8, 10]. Under free access to general miso soup, salt consumption varied so that it was technically difficult to match salt intake from common miso with that of the control saline. In the present study, we wanted to compare the effects of miso and a low-salt control on BP; hence, salt concentrations differed between the miso and control groups. Daily consumption of awase miso soup with 3.8 g of salt has been shown to increase SBP by 6.46 mmHg [1]. However, in our study, there was no increase in daytime casual BP.

Long-term awase miso intake does not influence body weight, glucose metabolism, or lipid metabolism. Common miso contains various nutrients. However, whether these nutrients work as expected when common miso is consumed is unclear. The present study shows that the nutrients in the miso combination were insufficient in terms of improving health or glucose or lipid metabolism in subjects without these abnormalities. On the other hand, serum creatinine levels were significantly lower in the miso group than in the control group. Since the serum creatinine level was lower in the miso than in the control group at baseline, it is not clear whether miso intake decreases serum creatinine levels, as reported in animal studies [5, 6].

In the present study, subjects with stage-1 hypertension also had a tendency toward decreased nighttime BP with miso intake. The miso might be more effective in subjects with high-normal BP. Otherwise, the subgrouping decreased the sample size (miso, 15 subjects; control, 14 subjects), and it decreased the statistical power to detect the differences. In fact, if we calculate the sample size needed to find the differences, > 35 subjects were needed for BP changes at 0200 hours and > 40 subjects for BP changes at 0400 hours (MedCalc Statistical Software).

In conclusion, we show that long-term awase miso intake does not influence daytime BP despite its relatively high salt concentration (3.8 g in two servings). It does, however, significantly reduce nighttime BP, perhaps by promoting diuresis. This is the first report to show that long-term awase miso intake decreases BP in humans.

### Terminology

Common miso: rice miso commercially available for daily cooking

Nenrin miso: registered rice miso commercially available (Mamukome Co., Ltd.) representative of common miso

MK-34-1 miso: new rice miso with ACE inhibitory activity from Mamukome (awase) miso or test miso2:1 combination of Nenrin and MK-34-1 miso

Rice miso: most commonly used miso manufactured from soybeans, malted rice, and salt

Wheat miso: miso manufactured from soybeans, malted wheat, and salt, which is used in part of the Kyushu area
